# Maternal exposure to low levels of corticosterone during lactation protects adult rat progeny against TNBS-induced colitis: A study on GR-mediated anti-inflammatory effect and prokineticin system

**DOI:** 10.1371/journal.pone.0173484

**Published:** 2017-03-07

**Authors:** Manuela Zinni, Anna Rita Zuena, Veronica Marconi, Carla Petrella, Ilaria Fusco, Chiara Giuli, Nadia Canu, Cinzia Severini, Maria Broccardo, Vassilia Theodorou, Roberta Lattanzi, Paola Casolini

**Affiliations:** 1 Department of Physiology and Pharmacology "Vittorio Erspamer", Sapienza University of Rome, Rome, Italy; 2 Institute of Cell Biology and Neurobiology, CNR, Rome, Italy; 3 Department of System Medicine, University of Rome "Tor Vergata", Rome, Italy; 4 INRA, EI-Purpan, UMR 1331 TOXALIM Neuro-Gastroenterology and Nutrition Team, Toulouse, France; Augusta University, UNITED STATES

## Abstract

The early phase of life represents a critical period for the development of an organism. Interestingly, early life experiences are able to influence the development of the gastrointestinal tract and the reactivity to colonic inflammatory stress. We recently demonstrated that adult male rats exposed to low doses of corticosterone during lactation (CORT-nursed rats) are protected against experimental colitis induced by the intracolonic infusion of 2,4,6-trinitrobenzenesulfonic acid (TNBS). Based on these interesting results, we wanted to better investigate which cellular actors could be involved in the protection of CORT-nursed rats from TNBS-induced experimental colitis. Therefore, in the present work, we focused our attention on different factors implicated in GR-mediated anti-inflammatory effect. To address this issue, colonic tissues, collected from control and CORT-nursed healthy animals and from control and CORT-nursed colitic rats, were processed and the following inflammatory factors were evaluated: the expression of (i) glucocorticoid receptors (GR), (ii) glucocorticoid-induced leucine zipper (GILZ), (iii) phospho-p65NF-κB, (iv) the pro-inflammatory cytokines IL-1β and TNF-α, (v) the prokineticins PK2 and PK2L and (vi) their receptors PKR1 and PKR2. We found that adult CORT-nursed rats, in comparison to controls, showed increased expression of colonic GR and reduced expression of pro-inflammatory molecules (IL-1β, TNF-α, PK2 and PK2L) in response to inflammatory colitis. The observed changes were associated with an increase in GILZ colonic expression and with a reduction in phospo-p65NF-κB colonic expression.

## Introduction

The inflammatory bowel diseases (IBDs) are chronic relapsing-remitting or progressive inflammatory conditions of the gastrointestinal tract (GI) [1]. Their aetiology implicates a complex interplay between genes and environmental factors [2,3], such as stress [4]. Some human studies have reported a positive association between exacerbation and relapse of IBD and stress [5–13], while others have shown a null association or a minor role of stress [14–19]. This literature disagreement can be mainly due to the great difficulty in establishing the kind of stress and how stress is perceived by the subject. On the contrary, animal studies that allow the standardisation of the experimental procedure (type, intensity and duration of stress) have shown a direct association between stress and susceptibility to experimental colitis [20–25].

The early phase of life is a critical period for neuroendocrine and behavioural development and any stress in this period can hesitate in long-term outcomes [26–29]. Interestingly, maternal separation occurring during the early postnatal period, influences the homeostasis of the GI tract and the vulnerability to stress-induced colitis, as demonstrated by higher susceptibility to dextran sulphate sodium (DSS)-induced colitis in adult rodents [24]. On the other hand, our previous studies [27,30] conducted in rats showed that offspring nursed by mothers with mild hypercorticosteronaemia develop the ability to better cope with different situations later in life. In this animal model, the drinking water of mother rats during lactation was supplemented with corticosterone (0.2 mg/ml) [27,31]. Maternal corticosterone is in equilibrium between blood and milk in rodents [30,31], and the hormone is easily absorbed by the GI tract of the pups, as the glucocorticoid permeability of the gut is very high in early postnatal life, up to 17–18 days of age [32]. Indeed, the progeny of these mothers (CORT-nursed rats), once adults, showed improved learning capabilities, reduced fearfulness in anxiogenic situations, a persistent hyporeactivity of the hypothalamus-pituitary-adrenal axis due to an increased number of glucocorticoid receptors (GR) in the hippocampus, and resistance to ischemic neuronal damage [27,30]. Moreover, we have recently demonstrated that adult CORT-nursed rats are protected against 2,4,6-trinitrobenzenesulfonic acid (TNBS)-induced experimental colitis [33]. In fact, colitic CORT-nursed rats showed an improvement in some indices of the pathology (loss of body weight and food intake, increased colonic myeloperoxidase (MPO) activity, and mast cell degranulation) with respect to colitic control animals (adult male rats whose mothers drank water without corticosterone during lactation).

Based on these interesting results, we wanted to better investigate which cellular actors could be involved in the protection of CORT-nursed rats from TNBS-induced experimental colitis. Therefore, in the present work, we focused our attention on some molecular factors linked to GR activation.

Glucocorticoids (GCs) activate the cytosolic glucocorticoid receptors. The ligand-receptor complex then translocates into the nucleus, where it interacts with the promoter regions of different genes. One of the earliest genes induced by GCs is the glucocorticoid-induced leucine zipper (GILZ), a dexamethasone-inducible gene that is rapidly and ubiquitously induced by GCs [34]. GILZ belongs to the transforming growth factor-β (TGF-β) stimulated clone 22 (TSC-22) family of proteins and mediates some of the immunomodulatory effects of GCs [34–36]. The immunomodulatory activity of GILZ involves a physical interaction (homo- and heterodimerisation) between GILZ and the transcription factors that regulate the expression of pro-inflammatory genes [34]. One of the targets of GILZ is the transcription nuclear factor κB (NF-κB) [34], a cytosolic protein implicated in the regulation of the function of immune system and of inflammatory processes. After activation, NF-κB translocates into the nucleus, where it induces the expression of genes encoding for adhesion molecules and pro-inflammatory cytokines [37] involved in the development of the inflammatory response. Studies have shown that GILZ physically interacts with the p65 subunit of NF-κB, preventing its activation and nuclear translocation [38,39]. Thus, through a direct interaction with the p65 subunit, GILZ inhibits NF-κB activation and reduces the expression of pro-inflammatory cytokines such as IL-1β and TNF-α. Several chemokines are among the mediators of innate immunity regulated by NF-κB during inflammation [40]. The Bv8/Prokineticins belong to a new family of chemokines comprehending the amphibian Bv8 and Bo8 (from the skin secretion of *Bombina variegata* and *Bombina orientalis*) and the mammalian prokineticin 2 (PK2) [41]. PK2 exerts its biological functions by binding two G-protein-coupled receptors called prokineticin receptor 1 (PKR1) and prokineticin receptor 2 (PKR2), with comparable affinity for both receptors. PK2 displays a major role in inflammatory pain and results strongly up-regulated in inflammatory processes associated with infiltrating cells [42–44]. A splice variant of PK2, containing 21 amino acid insert has been also identified and named PK2L. Interestingly, Marsango and collaborators demonstrated that NF-κB is involved in the regulation of Bo8 expression [45].

Overall, also considering that it has been demonstrated that GILZ transgenic mice (overexpressing GILZ) are less susceptible to DNBS-induced colitis as compared to wild-type animals [35], in the present investigation, we studied the effects of TNBS-induced colitis in adult CORT-nursed rats, focusing on the following different factors involved in inflammation: (i) GR, (ii) GILZ, (iii) phospho-p65NF-κB, (iv) the pro-inflammatory cytokines IL-1β and TNF-α, (v) the prokineticins PK2 and PK2L and (vi) their receptors PKR1 and PKR2.

## Materials and methods

### Ethics statement

As indicated in [33], all experimental procedures were carried out according to EU Directive 86/609/EEC and to Italian legislation on animal experimentation. The experimental protocols were also approved by the Local Animal Care and Use Committee of Institut National de la Recherche Agronomique (authorisation number MP/02/46/11/08).

### CORT-nursed animal model

Female Wistar rats (Charles River, Calco, Italy) weighing 280–320 g were mated and then housed individually. The day after birth, litters were culled to eight pups (four males and four females). Mothers of control rats were maintained on tap water, whereas mothers of CORT-nursed rats had *ad libitum* access to a solution of 0.2 mg/ml corticosterone 21-hemisuccinate from postnatal day 1 to postnatal day 21. Weaning was performed at 21 days of age, and animals were then housed three per cage. Three-month-old male CORT-nursed rats and their controls were used in this study. They were kept in a temperature-controlled room (21°C) and were allowed free access to water and food.

### Induction of experimental colitis and tissue collection

The first day of experiment (day 0), both controls and CORT-nursed male animals were housed one per cage until the day of sacrifice (day 4) and divided in two groups: 1) healthy rats, intracolonically infused with saline and 2) colitic rats, intracolonically infused with TNBS (2,4,6-trinitrobenzenesulphonic acid). Overnight fasted control and CORT-nursed rats were anesthetised by intraperitoneal injection of xylazine (0.6 mg/kg) and ketamine (120 mg/kg), and colitis was induced by an intra-colonic (IC) administration of TNBS at a dose of 30 mg/kg in 0.3 ml of 50% ethanol. Healthy rats were intracolonically infused with 0.3 ml of saline. TNBS and/or saline were infused through a silicone catheter introduced into the distal colon, 6 cm into the anus, as previously described [46]. On the fourth day after a single TNBS and/or saline instillation, all animals were euthanized by CO_2_ inhalation and, for each experimental group, colonic tissue was collected and stored at -80°C.

The current research is a progression of the previous study [33] in which we had n = 12 rats for each experimental group and, in order to avoid the litter effect, each litter contributed one or maximum two offspring per group. In the current study, we have analysed a total number of 17 samples coming from different litters, and divided as follows: control healthy (n = 4), control colitic (n = 5), CORT-nursed healthy (n = 4) and CORT-nursed colitic (n = 4). Of note, all samples were tested for an inflammatory state by myeloperoxidase (MPO) activity (see ref. 33 for technical details and results).

### RNA extraction and real-time PCR

Total RNA was extracted from whole distal colonic samples using the Trizol reagent (Invitrogen, Carlsbad, CA) according to the manufacturer’s instructions. RNA yield and purity were determined by spectrophotometric absorption at 260 and 280 nm. mRNA was quantified and 1 μg of mRNA was used to perform reverse transcription (Reverse Transcriptase, Promega) to obtain cDNA. cDNA was used as a template in real-time PCR (iCycler; Bio-Rad) using iQ SYBR Green Supermix (Bio-line). All the measures were performed in triplicate. The reaction conditions were as follows: 95°C for 10 min (polymerase activation), followed by 40 cycles at 95°C for 15 min, 55–50°C (temperature depends on the Tm of the primers) for 15 s and 72°C for 15 s. The reaction mixture without cDNA was used as the control. The results were quantified using the comparative threshold method. The Ct value of the specific gene of interest was normalised to the Ct value of the endogenous control, β-actin, and the comparative Ct method (2^-ΔΔCt^) was then applied using control healthy rats as the reference samples. The primer sequences are available in S1 Table.

### Western blotting analysis

Tissue from whole distal colonic samples from control or CORT-nursed rats was homogenised at 4°C in 200 μl of Ripa buffer (Sigma) containing protease inhibitors (1% v/v) (Sigma). Protein concentrations were determined using the Bradford protein assay. Forty micrograms of protein were re-suspended in sodium dodecyl sulphate (SDS)-bromophenol blue loading buffer with 0.7 M 2-mercaptoethanol. The samples were boiled for five minutes and separated on 8% (for GR), 10% (for PKR1 and PKR2) or 15% (for PK2) SDS-polyacrylamide gels. After electrophoresis (Bio-Rad, Mini Protean Tetra-Cell), the proteins were transferred to nitrocellulose membranes (Bio-Rad) using a system of mini transblot cell (Bio-Rad) at 4°C. After transfer, blots were incubated in a blocking solution containing Tris-buffered saline (TBS), 10% (w/v) Tween-20, 1% (w/v) non-fat milk and 1% (w/v) bovine serum albumin for GR, PKR1, PKR2, β-actin and in a solution containing Tris-buffered saline (TBS), 10% (w/v) Tween-20, 5% (w/v) non-fat milk for PK2. Subsequently, blots were incubated overnight with rabbit anti-GR (1:1000, sc-1004, Santa Cruz Biotechnology Inc.), goat anti-PKR1 (1:500, sc-54313, Santa Cruz Biotechnology Inc.), goat anti-PKR2 (1:500, sc-54313, Santa Cruz Biotechnology Inc.) and rabbit anti-PK2 (1:500; 87360, Abcam) in blocking solution at 4°C. After incubation with the primary antibody, the blots were incubated with horseradish peroxidase-conjugated goat anti-rabbit (1:2000; 7054, Cell Signaling), horseradish peroxidase-conjugated goat anti-mouse (1:2000; 7056, Cell Signaling) or with horseradish peroxidase-conjugated rabbit anti-goat (1:10000; 6741, Abcam) for 1 h at room temperature (21°C±2). To ensure that each lane was loaded with an equivalent amount of protein, the blots were probed with an anti-β actin serum (1:1000; Sigma) overnight at 4°C. Subsequently, blots were incubated with horseradish peroxidase-conjugated goat anti-mouse antibodies (1:2000; 7056, Cell Signaling) for 1 h at room temperature. Bands were visualised with an enhanced chemiluminescence system (Aurogene). After immunoblotting, digitised images of bands immunoreactive for target and control (actin) molecules were acquired and the area of immunoreactivity corresponding to each band was measured using the NIH ImageJ medical imaging software. A ratio of target to actin was then determined, and these values were compared for statistical significance.

### Immunofluorescence assay

Distal colonic samples were collected and fixed in 4% paraformaldehyde and immersed for 24 h in 30% of sucrose at 4°C. Samples were embedded Tissue-Tek OCT (Sakura Finetek Europe B.V) and frozen in isopentane at -45°C. Sections (8-μm thickness) were cut at -25°C and mounted directly on glass slides. Sections were then processed using fluorescent methods. Slides were pre-incubated with 3% normal goat serum in PBS + 0.3% Triton X-100 (PBST) for 2 h and then incubated overnight at 4°C with rabbit anti-GILZ (1:200, sc-33780, Santa Cruz) or rabbit anti-phospho-p65NF-κB (1:200, 3033S, Cell Signaling) dissolved in PBST + 1% goat serum. After washing in PBS, slides were incubated with a goat anti-rabbit rhodamine-conjugated secondary antibody (Sigma) for 30 min at room temperature. For nuclei visualisation, slides were incubated with Hoechst 33258 (0.25 μg/ml) for 5 min at room temperature. Sections were visualised by a confocal laser scanning microscope (Leica SP5, Leica Microsystems, Wetzlar, Germany), and the images captured using 40X oil objective. Pinhole size, image resolution, colour depth, scan speed and averaging (number of scans per image) were the same for all samples analysed. Immunofluorescence was quantified in three fields for every section in at least three sections for each sample by converting pixels in brightness values using the RGB (red, green and blue) [47]. The number of red cells is expressed as percentage (%) of positive labelled with respect to the total area (1024x1024 inches) with comparable blue Integrated Density Value (IDV).

### Statistical analysis

Results were analysed by two-way ANOVA (CORT treatment x colitis). The ANOVA analyses were followed by Fisher’s LSD *post-hoc* comparisons, where appropriate. Statistical significance was set at p<0.05. All data are expressed as sample mean ± SEM.

## Results

### Colonic GR expression

**Fig 1:**
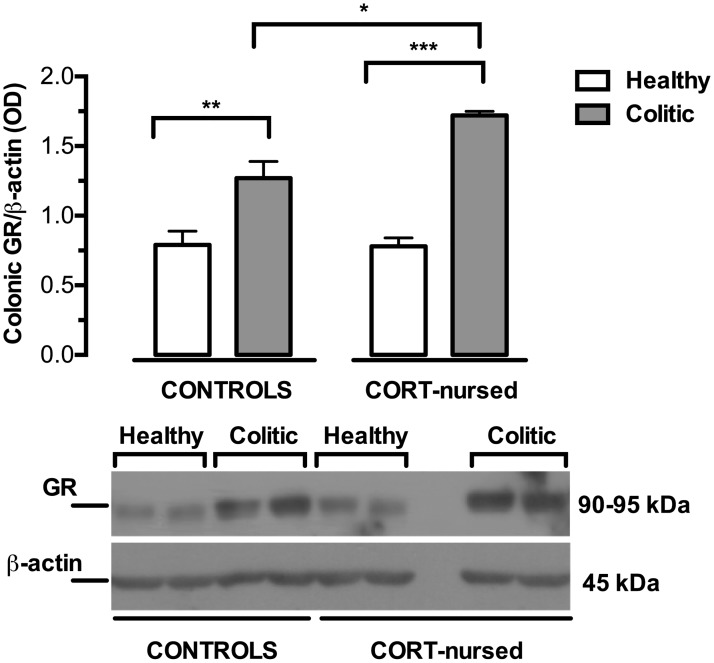
Effects of TNBS-induced colitis on colonic GR expression. Four days after TNBS instillation, increased colonic GR expression was observed in both control and CORT-nursed colitic animals (Fisher’s LSD *post-hoc*, **p<0.01, ***p<0.001 vs. own healthy animals). The CORT-nursed colitic group showed GR expression levels greater than those of the control colitic group (Fisher’s LSD *post-hoc*, *p<0.05). The results are expressed as the ratio of optical density (OD) of the GR to that of the β-actin band (control healthy n = 4, control colitic n = 5, CORT-nursed healthy n = 4, CORT-nursed colitic n = 4). A representative western blot image was reported.

As reported in Fig 1, two-way ANOVA (colitis x CORT treatment) showed a main effect of colitis (F_1,13_ = 59.72, p<0.0001) and a significant effect of CORT treatment (F_1,13_ = 5.73, p<0.05) on colonic GR expression. Moreover, the ANOVA analysis revealed a significant interaction between colitis and CORT treatment (F_1,13_ = 6.27, p<0.05). On this basis, we performed Fisher’s LSD *post-hoc* analysis to show that TNBS instillation significantly increased the colonic GR expression in control (p<0.01) and in CORT-nursed colitic animals (p<0.001), with respect to the corresponding healthy rats. Of note, the inflammatory colitis-induced increase in GR was greater in CORT-nursed than in control colitic animals (Fisher’s LSD *post-hoc*, p<0.05). No differences were observed between control and CORT-nursed healthy animals.

### Colonic GILZ immunofluorescent staining

**Fig 2:**
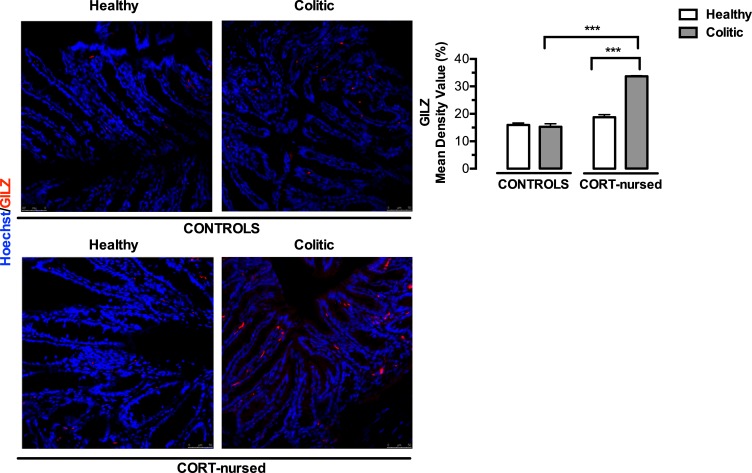
Effects of TNBS-induced colitis on colonic GILZ expression. Four days after TNBS instillation, an increase in colonic GILZ expression was observed in CORT-nursed colitic animals (Fisher’s LSD *post-hoc*, ***p<0.001 vs. CORT-nursed healthy). GILZ expression was significantly greater in CORT-nursed colitic rats with respect to control colitic rats (Fisher’s LSD *post-hoc*, ***p<0.001). Tissue sections were incubated with a primary antibody against GILZ (red signal). Nuclei were visualised by Hoechst 33258 (blue signal). Results are expressed as percentage (%) of positive labelled with respect to the total area (control healthy n = 4, control colitic n = 5, CORT-nursed healthy n = 4, CORT-nursed colitic n = 4). Representative immunofluorescence images are shown.

As reported in Fig 2, two-way ANOVA (colitis x CORT treatment) showed a significant effect of colitis (F_1,13_ = 65.96, p<0.0001) and of CORT treatment (F_1,13_ = 147.6, p<0.0001) on colonic GILZ expression. Furthermore, a significant interaction between colitis and CORT treatment was observed (F_1,13_ = 80.48, p<0.0001). Fisher’s LSD *post-hoc* analysis showed no difference between control colitic and control healthy animals and a significant increase in GILZ expression in the CORT-nursed group (CORT-nursed colitic vs. CORT-nursed healthy) (p<0.001). Interestingly, CORT-nursed colitic animals showed a greater colonic GILZ expression in comparison with control colitic group (Fisher’s LSD *post-hoc*, p<0.001). No differences were observed between control and CORT-nursed healthy animals.

### Colonic phospho-p65NF-κB levels

**Fig 3:**
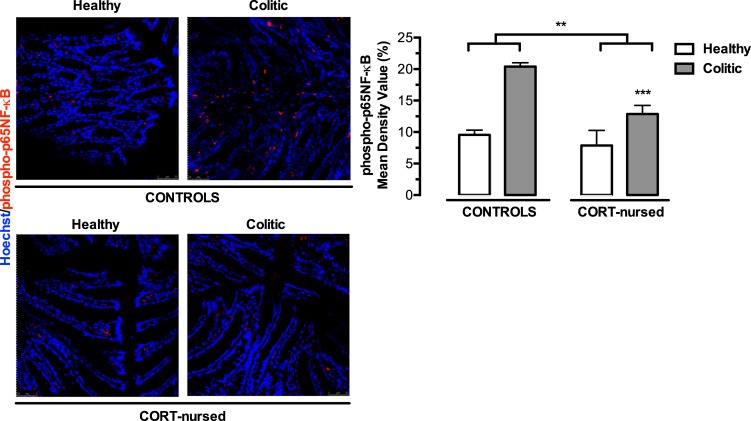
Effects of TNBS-induced colitis on colonic phospho-p65NF-κB expression. Four days after TNBS instillation, an increase in colonic phospo-p65NF-κB expression was observed in colitic animals (***p<0.001 colitic vs. healthy group). A reduction in phospo-p65NF- κB was observed in CORT-nursed rats (**p<0.01 CORT-nursed vs. control group). Tissue sections were incubated with a primary antibody against phospho-p65NF-κB (red signal). Nuclei were visualised by Hoechst 33258 (blue signal). Results are expressed as percentage (%) of positive labelled with respect to the total area (control healthy n = 4, control colitic n = 5, CORT-nursed healthy n = 4, CORT-nursed colitic n = 4). Representative immunofluorescence images are shown.

We next investigated the effect of GILZ overexpression showed by CORT-nursed rats, on NF-κB activation, through the expression of colonic levels of phosphorylated p65 (Fig 3). TNBS instillation significantly increased colonic phospho-p65NF-κB expression in colitic animals with respect to healthy rats (colitis effect, F_1,13_ = 32.30, p<0.001). Moreover, a significant reduction in NF-κB activation was observed in CORT-nursed rats with respect to control group (CORT treatment effect, F_1,13_ = 10.94, p<0.01). We did not found a clear evidence of non-linear interaction between CORT treatment and colitis (F_1,13_ = 4.39, p = 0.056) while a pure additive effect was observed.

### Colonic mRNA expression of the pro-inflammatory cytokines IL-1β and TNF-α

**Fig 4:**
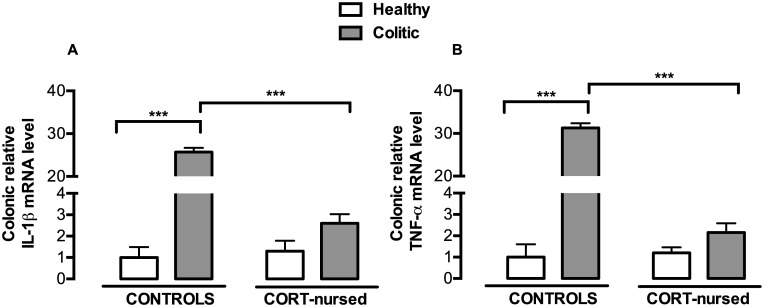
Effects of TNBS-induced colitis on colonic IL-1β and TNF-α mRNA expression. (A) An increase in colonic IL-1β mRNA expression was observed in control colitic rats (Fisher’s LSD *post-hoc*, ***p<0.001 vs. control healthy rats). A reduction in IL-1β mRNA expression was observed in CORT-nursed colitic rats (Fisher’s LSD *post-hoc*, ***p<0.001 vs. control colitic rats). (B) An increase in colonic TNF-α mRNA expression was observed in control colitic rats (Fisher’s LSD *post-hoc*, ***p<0.001 vs. control healthy rats). A reduction in TNF-α mRNA expression was observed in CORT-nursed colitic rats (Fisher’s LSD *post-hoc*, ***p<0.001 vs. control healthy rats). The colonic relative mRNA expression levels are expressed in relation to β-actin and presented as fold increases relative to control rats. (Control healthy n = 4, control colitic n = 5, CORT-nursed healthy n = 4, CORT-nursed colitic n = 4).

Two-way ANOVA (colitis x CORT treatment) showed a significant effect of colitis on IL-1β (F_1,13_ = 335.4, p<0.0001) and TNF-α (F_1,13_ = 422.2, p<0.0001) and a significant effect of CORT treatment on both cytokines expression (for IL-1β F_1,13_ = 257.9, p<0.0001; for TNF-α F_1,13_ = 362.3, p<0.0001) (Fig 4A and 4B). A significant interaction between CORT treatment and colitis was observed (for IL-1β F_1,13_ = 271.6, p<0.0001; for TNF-α F_1,13_ = 372.4, p<0.0001). Fisher’s LSD *post-hoc* analysis of the four experimental groups showed that cytokines expression followed the same trend as NF-κB activation. Indeed, inflammatory colitis induced a significant increase in the mRNA levels of IL-1β (Fig 4A) (p<0.001) and TNF-α (p<0.001) (Fig 4B) in control colitis rats, with respect to control healthy animals, while no modification of these pro-inflammatory cytokines was observed in CORT-nursed colitic rats, in comparison with CORT-nursed healthy rats. Moreover, CORT-nursed colitic rats had lower levels of IL-1β (Fisher’s LSD *post-hoc*, p<0.001) and TNF-α (Fisher’s LSD *post-hoc*, p<0.001) mRNA, with respect to control colitic animals. No differences were observed between control and CORT-nursed healthy animals.

### mRNA and protein expression of the colonic prokineticins PK2 and PK2L

**Fig 5:**
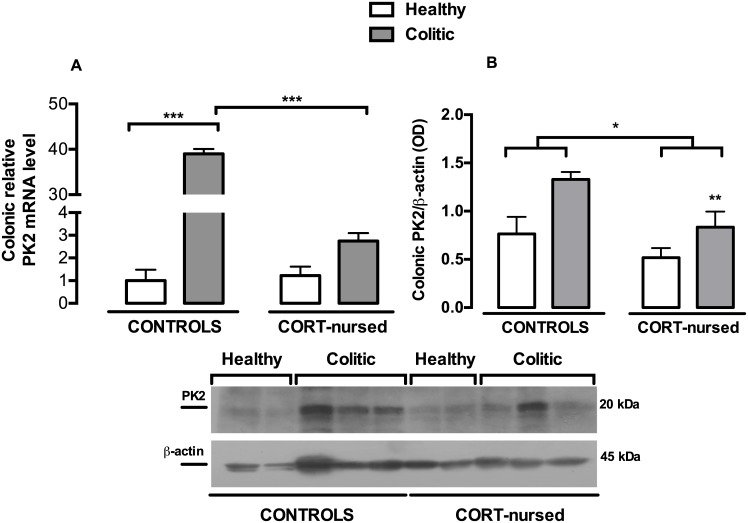
Effects of TNBS-induced colitis on colonic PK2 mRNA and protein expression. (A) Four days after TNBS instillation, an increase in colonic PK2 mRNA expression was found in control colitic rats (Fisher’s LSD *post-hoc*, ***p<0.001 vs. control healthy rats). A reduction in PK2 mRNA levels was observed in CORT-nursed colitic animals (Fisher’s LSD *post-hoc*, ***p<0.001 vs. control colitic rats). The colonic relative mRNA expression levels are expressed in relation to β-actin and presented as fold increase relative to control rats. (B) Western blot analysis showed an increase in PK2 in colitic rats (**p<0.01colitic vs. healthy group). A reduction in PK2 was observed in CORT-nursed rats (*p<0.05 CORT-nursed vs. control group). Results are expressed as the ratio of the optical density (OD) of the PK2 and the β-actin band. (control healthy n = 4, control colitic n = 5, CORT-nursed healthy n = 4, CORT-nursed colitic n = 4). A representative western blot image is shown.

**Fig 6:**
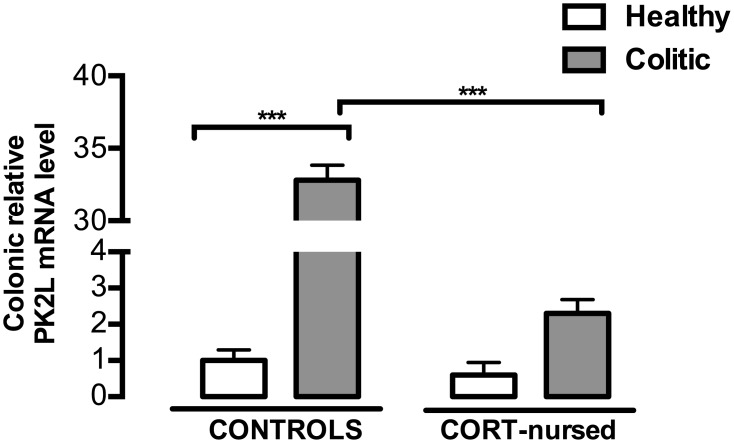
Effects of TNBS-induced colitis on colonic PK2L mRNA expression. Four days after TNBS instillation, an increase in colonic PK2L mRNA expression was revealed in control colitic rats (Fisher’s LSD *post-hoc* ***p<0.001 vs. control healthy rats). PK2L mRNA levels in CORT-nursed colitic animals were significantly lower (Fisher’s LSD *post-hoc* ***p<0.001 vs. control colitic). The colonic relative mRNA expression levels are expressed in relation to β-actin and presented as fold increase relative to control rats. (control healthy n = 4, control colitic n = 5, CORT-nursed healthy n = 4, CORT-nursed colitic n = 4).

Fig 5A shows the effect of colitis and CORT treatment on PK2 mRNA colonic expression. Two-way ANOVA (colitis x CORT treatment) showed a significant effect of colitis (F_1,13_ = 736.0, p<0.0001) and a significant effect of CORT treatment (F_1,13_ = 611.4, p<0.0001). An interaction between colitis and CORT treatment was observed (F_1,13_ = 626.5, p<0.0001). Fisher’s LSD *post-hoc* analysis showed a significant increase in PK2 mRNA in control colitic rats with respect to control healthy group (p<0.001), whereas no substantial difference was observed in CORT-nursed colitic animals, in comparison with CORT-nursed healthy rats. Interestingly, a significant reduction in PK2 mRNA was observed in CORT-nursed colitic rats, with respect to control colitic animals (p<0.001). No differences were found between control and CORT-nursed healthy animals. Concerning the PK2 protein expression (Fig 5B), TNBS instillation significantly increased colonic PK2 in colitic animals with respect to healthy rats (colitis effect, F_1,13_ = 11.50, p<0.01) and CORT treatment significantly reduced PK2 expression in CORT-nursed rats, with respect to control group (CORT treatment effect, F_1,13_ = 8.16, p<0.05). No significant interaction between CORT treatment and colitis was observed (F_1,13_ = 0.92, p = 0.35). Regarding the PK2 long isoform (PK2L) mRNA (Fig 6), two-way ANOVA showed a main effect of colitis (F_1,13_ = 594.2, p<0.0001), of CORT treatment (F_1,13_ = 505.6, p<0.0001) and an interaction between the two factors (F_1,13_ = 479.7, p<0.0001). In agreement with the PK2 mRNA results, we observed an increase in PK2L mRNA in control colitic rats with respect to control healthy animals (Fisher’s LSD *post-hoc*, p<0.001), while no difference was showed in CORT-nursed colitic animals with respect to CORT-nursed healthy rats. Interestingly, a significant reduction in PK2L mRNA was also observed in CORT-nursed colitic rats with respect to control colitic rats (Fisher’s LSD *post-hoc*, p<0.001). No differences were observed between control and CORT-nursed healthy animals. We could not evaluate PK2L protein levels because there are no antibodies available at the moment.

### mRNA and protein expression of the colonic prokineticin receptors PKR1 and PKR2

**Fig 7:**
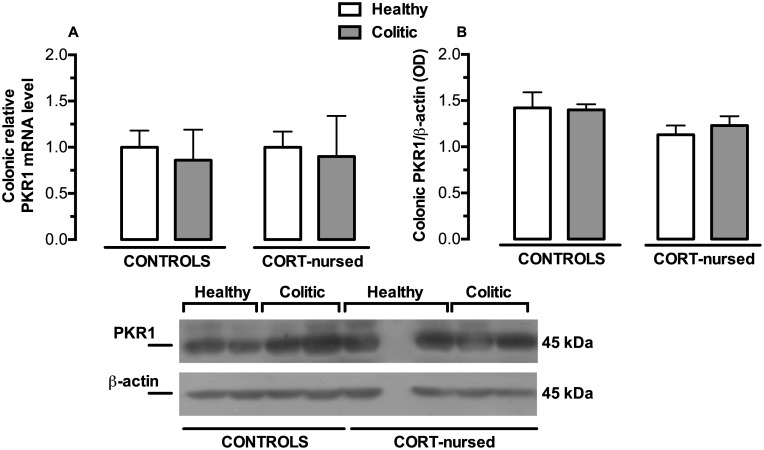
Effects of TNBS-induced colitis on colonic PKR1 mRNA and protein expression. (A) Four days after TNBS instillation, no significant differences were observed in colonic PKR1 mRNA expression between groups. The colonic relative mRNA expression levels are expressed in relation to β-actin and presented as fold increase relative to control rats. (B) Analysis of PKR1 protein expression showed no differences between experimental groups. Results are expressed as the ratio of the optical density (OD) of the PKR1 and the β-actin band. (control healthy n = 4, control colitic n = 5, CORT-nursed healthy n = 4, CORT-nursed colitic n = 4). A representative western blot image is shown.

**Fig 8:**
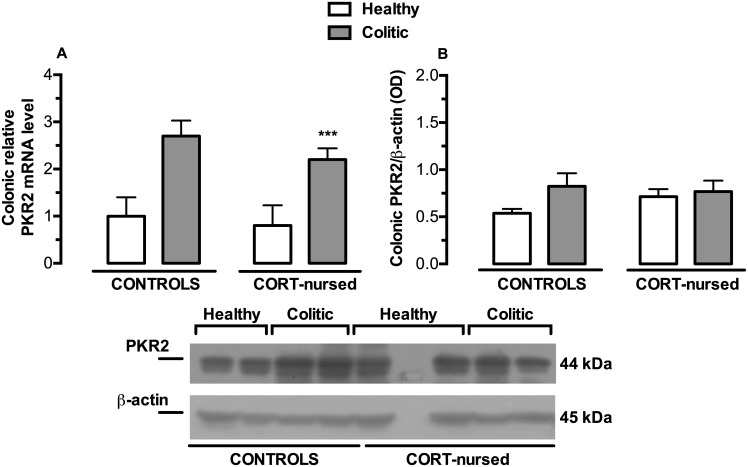
Effects of TNBS-induced colitis on colonic PKR2 mRNA and protein expression. (A) Four days after TNBS instillation, an increase in colonic PKR2 mRNA expression was observed in colitic rats (***p<0.001colitic vs. healthy group). The colonic relative mRNA expression levels are expressed in relation to β-actin and presented as fold increase relative to control rats. (B) Western blot analysis showed no differences between experimental groups. Results are expressed as the ratio of the optical density (OD) of the PKR2 and the β-actin band. (control healthy n = 4, control colitic n = 5, CORT-nursed healthy n = 4, CORT-nursed colitic n = 4). A representative western blot image is shown.

PKR1 mRNA and protein expression were not affected by colitis and of CORT treatment (Fig 7A and 7B). TNBS instillation significantly increased colonic PKR2 mRNA colonic expression in colitic animals with respect to healthy rats(Fig 8A), (colitis effect F_1,13_ = 18.77, p<0.001). On the contrary, no significant effect of CORT treatment and no significant interaction between the two factors were observed. No differences were found between control and CORT-nursed healthy animals and between control colitic and CORT-nursed colitic animals. PKR2 protein expression (Fig 8B) did not show differences between groups.

## Discussion

We recently demonstrated that CORT-nursed animals were protected against a TNBS-induced experimental colitis [33]. In fact, adult male offspring reared by dams that drank water supplemented with low dose of corticosterone during lactation (CORT-nursed rats) showed an improvement in some indices of pathology (loss of body weight and food intake, increased colonic MPO activity and mast cell degranulation) with respect to colitic control animals (adult male offspring whose mothers drank water during lactation). The results presented here indicate that CORT-nursed colitic rats showed an increase in colonic GR expression that in turn, triggers modifications of molecular factors implicated in GR intracellular anti-inflammatory response: GILZ over-expression, reduction in colonic NF-κB activation and reduced expression of the pro-inflammatory cytokines IL-1β and TNF-α and of the prokineticin PK2.

GR are expressed in different tissues, including the intestine, where they exert a role in controlling the local inflammatory state [48–50]. The results of present research show that TNBS-induced colitis increased colonic GR expression both in control and CORT-nursed rats. Interestingly, this increase was more evident in the CORT-nursed group. In our previous study [33], on the contrary, we observed no change in colonic mucosa GR expression in colitic rats. The disagreement could be explained considering that, in the previous study, we analysed only the mucosal layer while in the present study we have analysed the whole colonic tissue, including both mucosal layers and all other colonic layers (submucosa, muscular layer and serosa). Therefore, the increased GR expression we observed may be accounted for colonic layers rather than by mucosal layer.

In inflammatory colitis, GCs exert some of their immunomodulatory effects by inducing the early gene GILZ [34]. In the present study, we demonstrated that CORT-nursed colitic rats, with respect to the control colitic group, showed higher colonic GILZ expression, which could contribute to lower susceptibility to the TNBS-induced colitis insult. This observation is well-supported by a study conducted in mice by Cannarile and collaborators, showing that GILZ transgenic mice (overexpressing GILZ) were less susceptible to a DNBS-induced colitis, as compared to wild-type animals [35]. Moreover, it has recently been demonstrated that a deficiency in GILZ in knock-out mice exacerbates the effects of chemically-induced colitis [51].

One way by which GILZ could mediate its immunomodulatory effects is through binding to the p65 subunit of the transcription factor NF-κB [38,39]. This physical interaction prevents NF-κB activation and its nuclear translocation [38,39]. Our results demonstrate that protected CORT-nursed colitic rats showed reduced activation of NF-κB, expressed as a reduced level of colonic phospho-p65NF-κB. Therefore, sustained by the study conducted by Cannarile and collaborators [35], we can hypothesise that GILZ overexpression reduces NF-κB activation induced by intracolonic TNBS administration and that GILZ-dependent inhibition of the NF-κB pathway could contribute to the lower susceptibility to TNBS-induced colitis observed in CORT-nursed rats. However, the induction of GILZ expression is not the only mechanism by which GCs can exert their anti-inflammatory action. Indeed, activated GR are also able to interact directly with NF-κB with a consequent inhibition of NF-κB translocation and activity [52]. Thus, we cannot exclude that the protection observed in CORT-nursed rats could also be ascribed to the direct inhibition of NF- κB promoted by the GR itself.

After translocation into the nucleus, NF-κB induces the expression of pro-inflammatory genes such as those encoding for adhesion molecules and pro-inflammatory cytokines [37]. The results presented here indicate that, in CORT-nursed rats, in comparison with control colitic animals, the colonic pro-inflammatory cytokine levels are in line with the NF-κB activation profile: a reduction in NF-κB activation corresponded to a reduction in IL-1β and TNF-α mRNA levels. Since NF-κB-mediated cytokine release plays a critical role in the development of experimental colitis [53], the present data suggest that the inhibitory role of GILZ on NF-κB activity in colonic cells, mediated by GR activation, could contribute to the prevention of disease development in CORT-nursed rats, through the inhibition of pro-inflammatory cytokine release. These results are again sustained by those of Cannarile and collaborators who demonstrated in GILZ overexpressing transgenic mice that the reduced susceptibility to DNBS colitis is associated with the inhibition of NF-κB activation and the resulting reduction in IL-1β and TNF-α expression [35].

During inflammatory processes, cytokines are released together with other molecules such as chemokines belonging to the Bv8/prokineticin family [41]. Here, we have shown that inflammatory colitis induced a significant increase in colonic PK2 mRNA levels and protein expression in control colitic animals, with respect to control healthy rats. The increase in PK2 under inflammatory conditions is well-demonstrated in different animal models of inflammation [42–44]. In particular, in agreement with our results, Watson and collaborators also showed that TNBS instillation increases colonic PK2 levels in rats [54]. Interestingly, in the less vulnerable CORT-nursed rats, PK2 (both mRNA and protein) levels were significantly reduced with respect to the control colitic rats. This protection could be ascribed to the further involvement of NF-κB with the prokineticin system. Indeed, it has been demonstrated that NF-κB is able to regulate the expression of the Bo8 gene and that the consensus sequence for NF-κB identified in the Bo8 promoter is similar to those present in the promoter regions of human and mouse homologs [45]. As reported for PK2, we have also observed an increase in PK2L (the PK2 long form) mRNA levels in colitic rats, confirming that PK2L strongly contributes to inflammation. Indeed, our previous work [43] demonstrated that, in an animal model of inflammation induced by the injection of complete Freund’s adjuvant in the hind paw, stronger inflammatory responses observed in rats correlate with higher PK2L mRNA expression in the rat paw. We suppose that PK2L strongly contributes to inflammation through the smaller peptide, PK2β, derived by its proteolytic cleavage. Indeed, in functional and receptor binding assays, the recombinant PK2L protein demonstrated very poor activity, whereas PK2β retained its activity and selective affinity for PKR1 [55]. As we observed a significant decrease in PK2L levels in CORT-nursed colitic rats, with respect to control colitic animals, we can assume that PK2L expression, similar to PK2 expression, could undergo regulation by NF-κB.

Regarding the prokineticin receptors, we found no differences in PKR1 levels (at both the mRNA and protein levels) between control and CORT-nursed colitis rats, while PKR2 mRNA was increased after TNBS-induced colitis; this is in accordance with previous evidence suggesting that only PKR2 is the inducible receptor [42,56]. Conversely, PKR2 protein expression levels were unchanged. Since we analysed PKR2 mRNA and protein expression at only one time point (four days after TNBS instillation), without performing a time-course, we cannot exclude the possibility that PKR2 mRNA up-regulation had just started at this time point and that increased protein expression occurred at a later time.

In summary, in the current research, we identified different factors implicated in GR-mediated anti-inflammatory effect that could be involved in the protection from colitis in CORT-nursed rats. However, we are aware of the limited number of samples we had at our disposal and of the limits of immunofluorescence analysis for the quantification of GILZ and NF-κB protein expression, so further studies are needed in order to better clarify the role of these factors in the lower susceptibility of CORT-nursed rats to colitis.

In conclusion, a question still arises: how does maternal exposure to a low dose of corticosterone during lactation protect the adult offspring from intestinal inflammation? Increased maternal plasma levels of corticosterone may be responsible for the effects in the offspring through two different hypothetic mechanisms: (i) by acting directly on the pups or (ii) by influencing maternal behaviour. Regarding the first point, maternal corticosterone reaching the pups through milk [31] might contribute to the induction of long-lasting changes in the gastrointestinal tract, thus modifying (in a protective way) parameters involved in the inflammatory gastrointestinal response. On the other hand, we demonstrated that hypercoticosteronaemic dams showed an increase in maternal care (arched-back nursing and licking/grooming behaviour) [30]. Thus, we cannot exclude that the positive modifications of inflammatory parameters could have been induced by maternal behaviour that, as demonstrated by Meaney, is able to affect gene expression through epigenetic mechanisms [57]. Certainly, the two mechanisms may coexist; thus, the protection of CORT-nursed rats against inflammatory colitis could be promoted by both maternal behaviour and corticosterone action.

## Supporting information

S1 Table Real-time PCR primer sequences. (DOCX)
