# Supplementary material for: Maternal exposure to low levels of corticosterone during lactation protects adult rat progeny against TNBS-induced colitis: A study on GR-mediated anti-inflammatory effect and prokineticin system
Source: PLoS One. 2017 Mar 7;12(3):e0173484. doi: 10.1371/journal.pone.0173484 (PMC5340375; doi:10.1371/journal.pone.0173484)
Supplement: S1 Table — (DOCX) [file pone.0173484.s001.docx]

| **TNF-α** | Forward: 5'-TCCCAACAAGGAGGAGAAGTTCC-3' |
| --- | --- |
|  | Reverse: 5'-GGCAGCCTTGTCCCTTGAAGAGA-3' |
| **IL-1β** | Forward: 5'-GCACCTTCTTTTCCTTCATCTTTG-3' |
|  | Reverse: 5'-TTTGTCGTTGCTTGTCTCTCCTT-3' |
| **PK2** | Forward: 5'-TCATCACCGGGGCTTGCG-3' |
|  | Reverse: 5'-TAACTTTCCGAGTCAGGG-3' |
| **PK2L** | Forward: 5'-AGGAAAGAAGAAGGGCGAAG-3' |
|  | Reverse: 5'-TCCTTAAACATGCCAAACCTG-3' |
| **PKR1** | Forward: 5'-CGCACCGTCTCCCTCTAC -3' |
|  | Reverse: 5'- GTTTGACACTTCATCCGCG-3' |
| **PKR2** | Forward: 5'-CTCCGTCAACTACCTTCGTA -3' |
|  | Reverse: 5'-GAGGCGGTCTGGTAATTCA-3' |
| **β-actin** | Forward: 5'-AGATGACCCAGATCATGTTTG-3' |
|  | Reverse: 5'-TAGATGGGCACAGTGTGG-3' |

**TNF-α:** tumor necrosis factor **α**; **IL-1β:** interleuchin-1 **β**; **PK2**: prokineticin 2; **PK2L**: prokineticin 2 long isoform; **PKR1:** prokineticin receptor 1; **PKR2:** prokineticin receptor 2; **β-actin: β**-actina
